# Supplementary material for: Microbial Diversity Impacts Non-Protein Amino Acid Production in Cyanobacterial Bloom Cultures Collected from Lake Winnipeg
Source: Toxins (Basel). 2024 Mar 26;16(4):169. doi: 10.3390/toxins16040169 (PMC11053616; doi:10.3390/toxins16040169)
Supplement: Supplementary file 1 [file toxins-16-00169-s001.zip › toxins-2895593-SU.pdf]

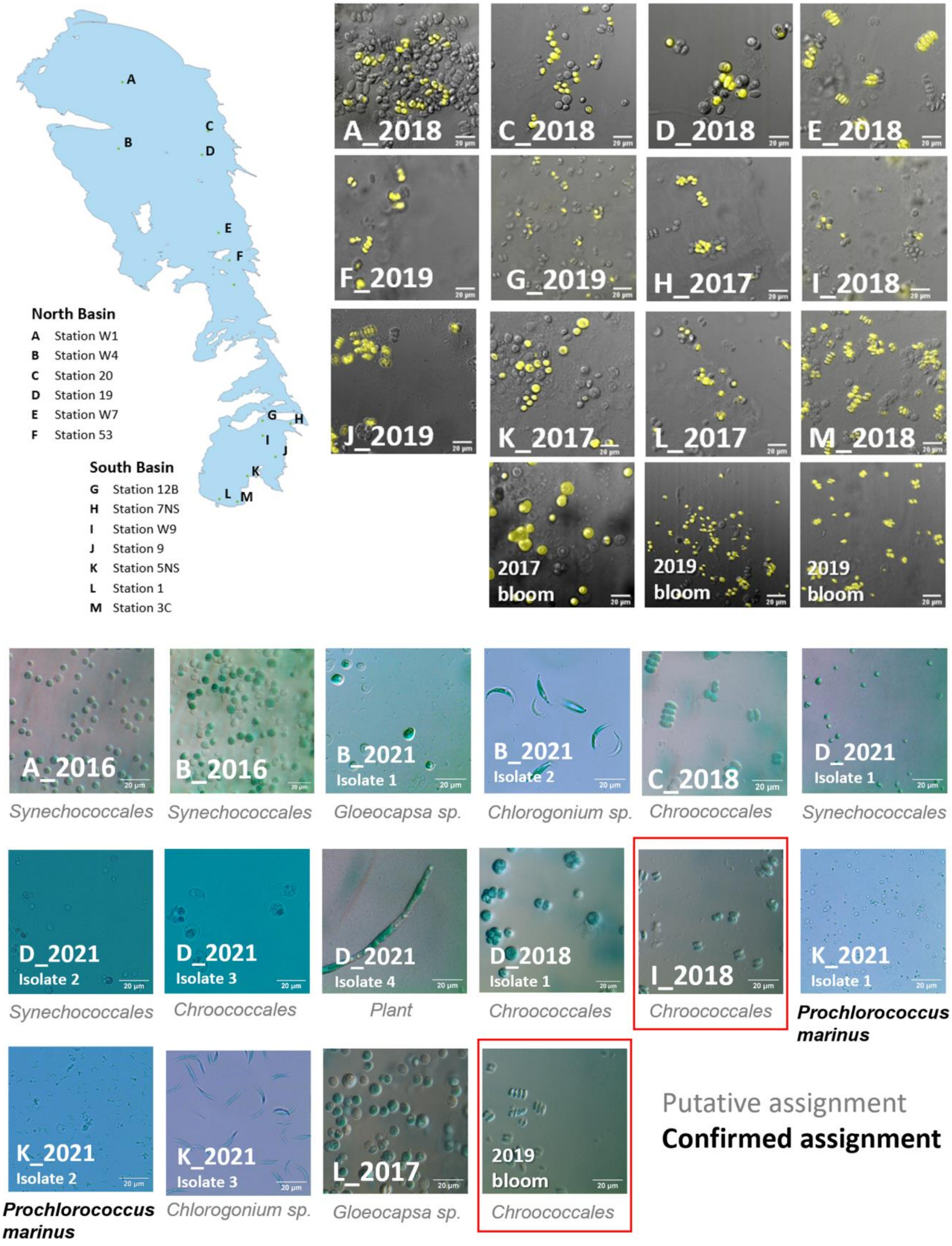


**Figure S1.** Microscopic images of cyanobacterial cultures. 40X magnification images of crude (greyscale images, cyanobacteria highlighted in yellow) and isolate (color images) cyanobacteria. Putative assignments based on morphological characteristics; confirmed assignments based on 16S rRNA taxonomic identification. Squares encapsulate putatively identical cyanobacterial species.

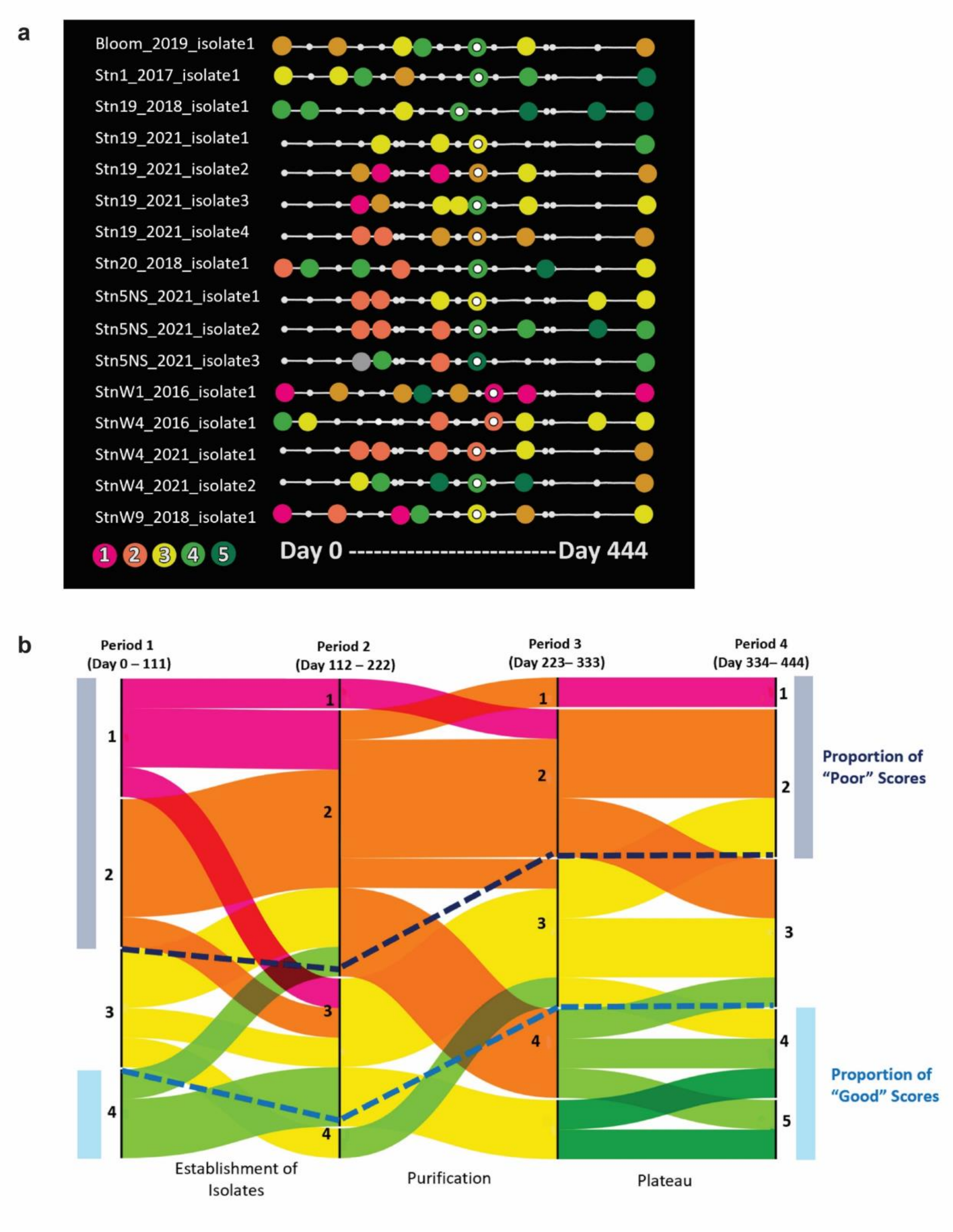


**Figure S2.** Culture quality tracking spanning a period of 445 days. (a) The lines are normalized to dates of subculturing to show progression over time. The colors used correspond to the qualitative 1-5 ranking. Grey indicates that a subculture was created, but no growth of any kind was observed. White circle denotes subculture used for non-protein amino acid quantification and 16S rRNA metagenomic sequencing. (b) Alluvial diagram showing median culture ranking during four 111 day periods over the course of the cyanobacterial culture purification process. "Good" scores defined as qualitative rankings of 4 or 5; "Poor" scores defined as qualitative rankings of 1 or 2.

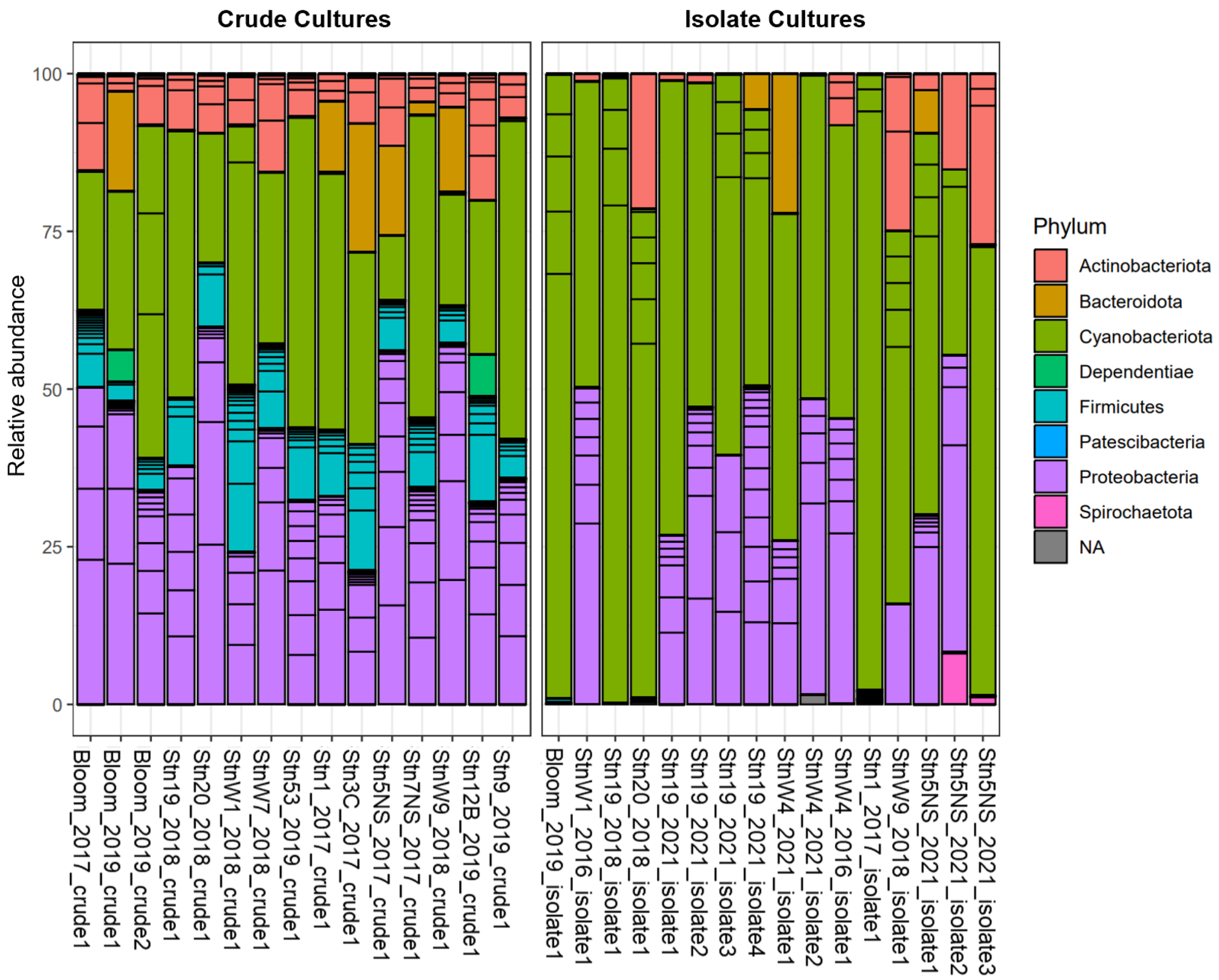


**Figure S3.** Metagenomic profiles of crude and isolate cultures. Profiles were computed using the DADA2 bioinformatic pipeline and taxonomy assigned using SILVA v138.1 and CyanoSeq v1.2 databases (bootstrap level = 50).

**Table S1.** Total β-*N*-methylamino-L-alanine (BMAA), 2,4-diaminobutyric acid (DAB), *N*-(2-aminoethyl)glycine (AEG) and β-aminomethyl-L-alanine (BAMA) concentrations for all bloom and isolate cyanobacterial cultures analyzed in this study, including collection sites and years. Values provided as averages ± standard error of the mean. ND means the compound was not detected in any replicates of the sample.

| Lake Section | Location | Year Collected | Culture Type | BMAA (ng/g) | | AEG (ng/g) | | DAB (µg/g) | | BAMA (ng/g) | |
|---|---|---|---|---|---|---|---|---|---|---|---|
| **Blooms** | Bloom | 2017 | Crude | ND | | 120.36 ± | 11.92 | 2.02 ± | 0.25 | 47.38 ± | 15.24 |
| | | 2019 | Crude | ND | | 108.75 ± | 5.28 | 0.41 ± | 0.03 | 37.54 ± | 16.71 |
| | | | Crude | ND | | 141.08 ± | 6.60 | 0.82 ± | 0.06 | 34.12 ± | 21.71 |
| | | | Isolate | 15.18 ± | 11.02 | 23.73 ± | 8.20 | 0.31 ± | 0.06 | 9.95 ± | 4/13 |
| **North Basin** | North Basin | 2018 | Crude | 347.39 ± | 45.19 | 104.97 ± | 3.17 | 3.65 ± | 0.29 | 35.82 ± | 16.82 |
| | | | Crude | 250.87 ± | 27.51 | 55.19 ± | 9.78 | 8.33 ± | 0.92 | 48.42 ± | 22.89 |
| | Stn 19 | 2018 | Crude | 96.60 ± | 22.35 | 52.56 ± | 6.00 | 0.71 ± | 0.04 | 17.11 ± | 2.60 |
| | | | Isolate | 45.90 ± | 5.90 | 23.73 ± | 9.93 | 0.38 ± | 0.10 | 19.40 ± | 3.46 |
| | | 2021 | Isolate 1 | 33.48 ± | 11.23 | 17.54 ± | 8.20 | 0.62 ± | 0.04 | 74.76 ± | 26.09 |
| | | | Isolate 2 | 49.84 ± | 15.89 | 38.49 ± | 15.39 | 1.21 ± | 0.10 | 71.94 ± | 58.45 |
| | | | Isolate 3 | ND | | 26.89 ± | 10.57 | 0.28 ± | 0.02 | ND | |
| | | | Isolate 4 | 19.55 ± | 5.66 | 27.96 ± | 4.93 | 0.15 ± | 0.02 | 86.80 ± | 10.00 |
| | Stn 20 | 2018 | Crude | 331.04 ± | 13.03 | 56.83 ± | 2.98 | 2.04 ± | 0.17 | ND | |
| | | | Crude | 317.64 ± | 25.83 | 111.68 ± | 7.39 | 8.35 ± | 0.55 | 25.74 ± | 9.06 |
| | | | Isolate | ND | | 18.17 ± | 8.44 | 0.80 ± | 0.23 | 6.32 ± | 2.73 |
| | Stn W1 | 2016 | Isolate | 73.13 ± | 35.07 | 53.97 ± | 19.82 | 0.36 ± | 0.05 | ND | |
| | | 2018 | Crude | 182.38 ± | 40.25 | 69.28 ± | 3.70 | 1.09 ± | 0.03 | ND | |
| | | | Crude | ND | | 54.63 ± | 4.05 | 0.97 ± | 0.10 | 11.64 ± | 4.67 |
| | Stn W4 | 2016 | Isolate | ND | | 42.97 ± | 17.72 | 0.11 ± | 0.02 | 188.01 ± | 97.30 |
| | | 2021 | Isolate 1 | 6.69 ± | 1.60 | 14.40 ± | 4.16 | 0.11 ± | 0.01 | 57.53 ± | 50.47 |
| | | | Isolate 2 | ND | | 16.65 ± | 5.02 | 0.50 ± | 0.02 | 26.28 ± | 18.63 |
| **North Basin Narrows** | Stn 53 | 2019 | Crude | ND | | 127.36 ± | 9.07 | 4.08 ± | 0.34 | 51.46 ± | 11.95 |
| | | | Crude | ND | | 103.55 ± | 9.25 | 0.36 ± | 0.03 | 22.47 ± | 7.64 |
| | Stn W7 | 2018 | Crude | 399.84 ± | 48.67 | 428.68 ± | 15.33 | 1.45 ± | 0.07 | 38.00 ± | 2.66 |
| | | | Crude | 279.34 ± | 42.49 | 42.42 ± | 5.07 | 1.59 ± | 0.09 | ND | |
| **South basin** | Stn 1 | 2017 | Crude | 127.01 ± | 20.68 | 35.07 ± | 1.96 | 3.79 ± | 0.43 | 13.79 ± | 5.61 |
| | | | Isolate | 16.16 ± | 4.26 | 20.64 ± | 12.51 | 0.23 ± | 0.01 | 38.51 ± | 29.35 |
| | Stn 12B | 2019 | Crude | ND | | 120.93 ± | 7.72 | 5.36 ± | 0.43 | 21.67 ± | 8.08 |
| | | | Crude | ND | | 176.22 ± | 11.84 | 4.30 ± | 0.32 | 21.05 ± | 4.18 |
| | Stn 3C | 2017 | Crude | 483.96 ± | 52.87 | 50.82 ± | 5.44 | 1.89 ± | 0.12 | 59.06 ± | 24.75 |
| | Stn 5 NS | 2017 | Crude | 113.41 ± | 21.30 | 193.55 ± | 18.94 | 2.72 ± | 0.26 | 31.98 ± | 10.48 |
| | | | Crude | 26.78 ± | 16.86 | 68.45 ± | 4.19 | 1.57 ± | 0.17 | 13.94 ± | 2.14 |
| | | 2021 | Isolate 1 | ND | | 34.10 ± | 10.27 | 4.78 ± | 0.73 | 39.80 ± | 9.63 |
| | | | Isolate 2 | ND | | 247.66 ± | 53.37 | 11.02 ± | 1.42 | 67.41 ± | 37.98 |
| | | | Isolate 3 | ND | | 43.01 ± | 17.04 | 18.65 ± | 2.37 | 84.78 ± | 34.13 |
| | Stn 7 NS | 2017 | Crude | 465.18 ± | 61.80 | 92.49 ± | 5.26 | 1.97 ± | 0.23 | 82.97 ± | 27.14 |
| | | | Crude | 139.19 ± | 20.82 | 28.69 ± | 0.34 | 1.27 ± | 0.08 | 28.04 ± | 17.12 |
| | Stn 9 | 2019 | Crude | ND | | 369.39 ± | 26.25 | 5.14 ± | 0.44 | 47.36 ± | 18.49 |
| | Stn W9 | 2018 | Crude | 206.43 ± | 13.96 | 94.78 ± | 8.41 | 1.67 ± | 0.16 | 21.65 ± | 6.34 |
| | | | Crude | ND | | 156.68 ± | 12.91 | 1.08 ± | 0.14 | 38.89 ± | 7.40 |
| | | | Isolate | 41.65 ± | 11.94 | 35.92 ± | 11.75 | 0.69 ± | 0.04 | 23.61 ± | 14.13 |
| | | 2019 | Crude | ND | | 77.58 ± | 2.05 | 2.91 ± | 0.27 | 25.01 ± | 11.62 |
| | | | Crude | ND | | 680.98 ± | 49.53 | 1.92 ± | 0.13 | 54.98 ± | 21.69 |

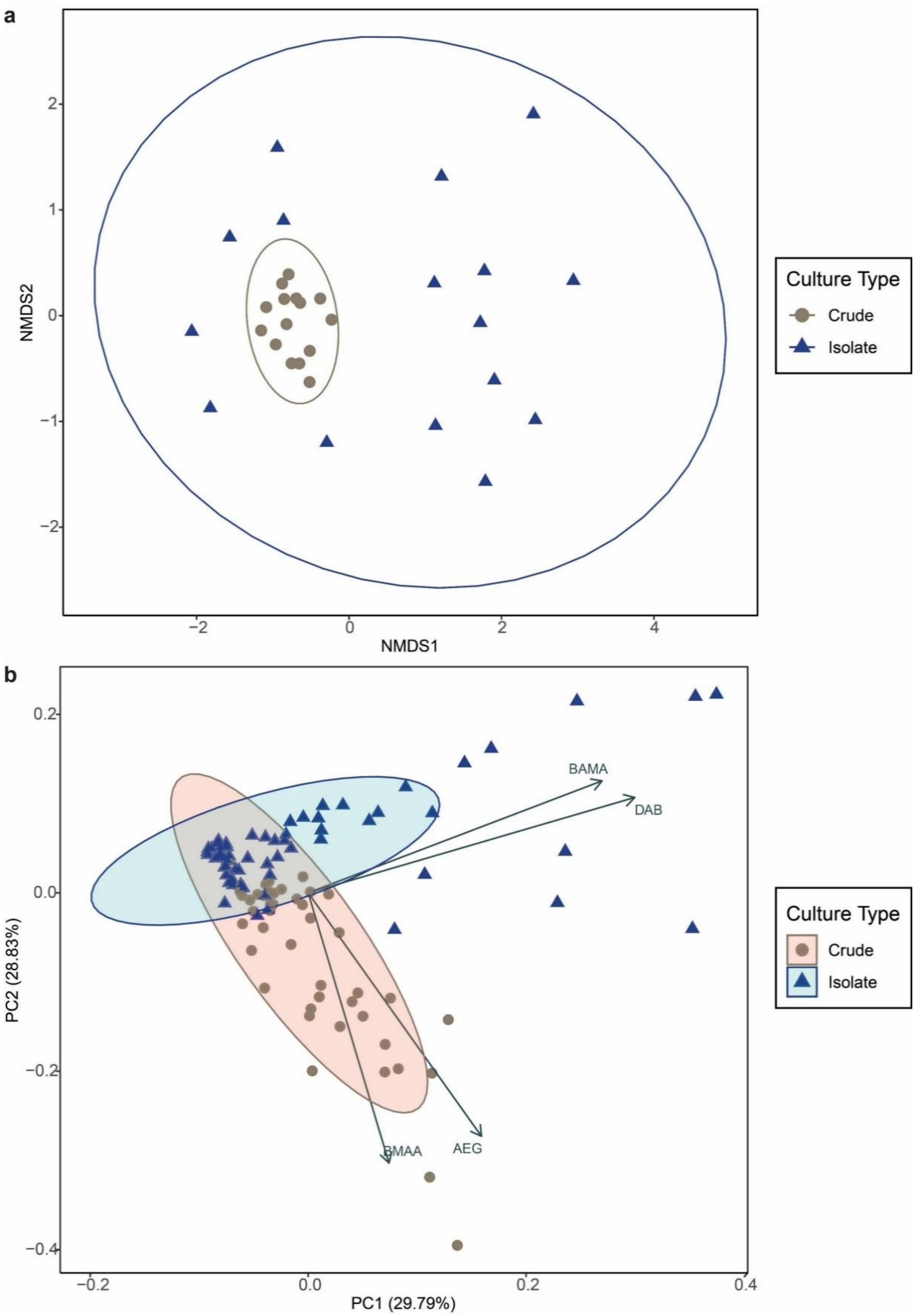


**Figure S4.** Correlations of non-protein amino acid (NPAA) concentrations to culture type. (a) Non-metric Multi-dimensional Scaling (NMDS) plot of beta diversity measure (Bray-Curtis distances) and (b) Principal Component (PC) Analysis biplot of auto-scaled NPAA concentrations showing clustering of crude cultures (brown circles) compared to isolate cultures (blue triangles). Ellipses show multivariate t-distribution at the 95% confidence level.

**Table S2.** Multiple reaction monitoring (MRM) transitions for validated ultra-performance liquid chromatography-tandem mass spectrometry (UPLC-MS/MS) method with AccQ-Fluor reagent.

| Analyte | Electrospray Ionization Mode | Retention Time (RT; min) | % Relative Standard Deviation (RT) | Transition (m/z) | Cone Voltage (V) | Collision Voltage (V) |
|---|---|---|---|---|---|---|
| β-aminomethyl-L-alanine (BAMA) | + | 5.56 | 0.60% | 459 > 171 | 16 | 30 |
| | | | | 459 > 119 | 16 | 18 |
| | | | | 459 > 289 | 16 | 15 |
| *N*-(2-aminoethyl)glycine (AEG) | + | 5.98 | 0.21% | 459 > 171 | 16 | 30 |
| | | | | 459 > 119 | 16 | 18 |
| | | | | 459 > 289 | 16 | 15 |
| | | | | 459 > 214 (qualifier) | 16 | 20 |
| β-*N*-methylamino-L-alanine (BMAA) | + | 6.24 | 0.45% | 459 > 171 | 16 | 30 |
| | | | | 459 > 119 | 16 | 18 |
| | | | | 459 > 289 | 16 | 15 |
| | | | | 459 > 258 (qualifier) | 16 | 20 |
| 2,4-diaminobutyric acid (DAB) | + | 6.55 | 0.22% | 459 > 171 | 16 | 30 |
| | | | | 459 > 119 | 16 | 18 |
| | | | | 459 > 289 | 16 | 15 |
| | | | | 459 > 188 (qualifier) | 16 | 20 |
